# Anisotropic impedance surfaces activated by incident waveform

**DOI:** 10.1515/nanoph-2021-0659

**Published:** 2022-02-07

**Authors:** Haruki Homma, Muhammad Rizwan Akram, Ashif Aminulloh Fathnan, Jiyeon Lee, Christos Christopoulos, Hiroki Wakatsuchi

**Affiliations:** Department of Engineering, Graduate School of Engineering, Nagoya Institute of Technology, Nagoya, Aichi, 466-8555, Japan; Electrical and Computer Engineering Department, University of California San Diego, La Jolla, CA, 92093, USA; The George Green Institute of Electromagnetics Research, Department of Electrical and Electronic Engineering, University of Nottingham, Nottingham, NG7 2RD, UK; Precursory Research for Embryonic Science and Technology (PRESTO), Japan Science and Technology Agency (JST), Saitama 332-0012, Japan

**Keywords:** anisotropic impedance surfaces, nonlinear circuit, power dependency, waveform selectivity

## Abstract

Anisotropic impedance surfaces have been used to control surface wave propagation, which has benefited applications across a variety of fields including radio-frequency (RF) and optical devices, sensing, electromagnetic compatibility, wireless power transfer, and communications. However, the responses of these surfaces are fixed once they are fabricated. Although tunable impedance surfaces have been introduced by utilizing power-dependent nonlinear components, such a tuning mechanism is generally limited to specific applications. Here we propose an additional mechanism to achieve tunable anisotropic impedance surfaces by embedding transient circuits that are controllable via the type of incident waveform. By switching between the open and short states of the circuits, it is possible to separately control the unit-cell impedances in two orthogonal directions, thereby changing from an isotropic impedance surface to an anisotropic impedance surface. Our simulation results show that a short pulse strongly propagates for both *x* and *y* directions at 3 GHz. However, when the waveform changes to a continuous wave, the transmittance for *x* direction is reduced to 26%, although still the transmittance for *y* direction achieves 77%. Therefore, the proposed metasurfaces are capable of guiding a surface wave in a specific direction based on the incident waveform even with the same power level and at the same frequency. Our study paves new avenues regarding the use of surface wave control in applications ranging from wireless communications to sensing and cloaking devices.

## 1 Introduction

Surface waves are waves bound with a surface, and they decay exponentially the farther away they are from the surface plane [1]. These waves are excited, for example, in response to external near-field radiation or because of nearby antennas. Oftentimes, these waves are undesirable as they can affect far-field radiation patterns, couple to nearby radiators, or produce interference that affects wireless or optical communications. However, precisely controlling these waves can be utilized in various applications, for instance, to design leaky-wave antennas [2, 3] or integrated plasmonic devices [4–6], to avoid undesirable coupling [7, 8], to nullify the surface wave in the substrate underneath the antenna [1], to channel all the energy in a given direction for wireless power transfer [9], or to feed an arbitrary antenna element among others [10].

**Figure 1:**
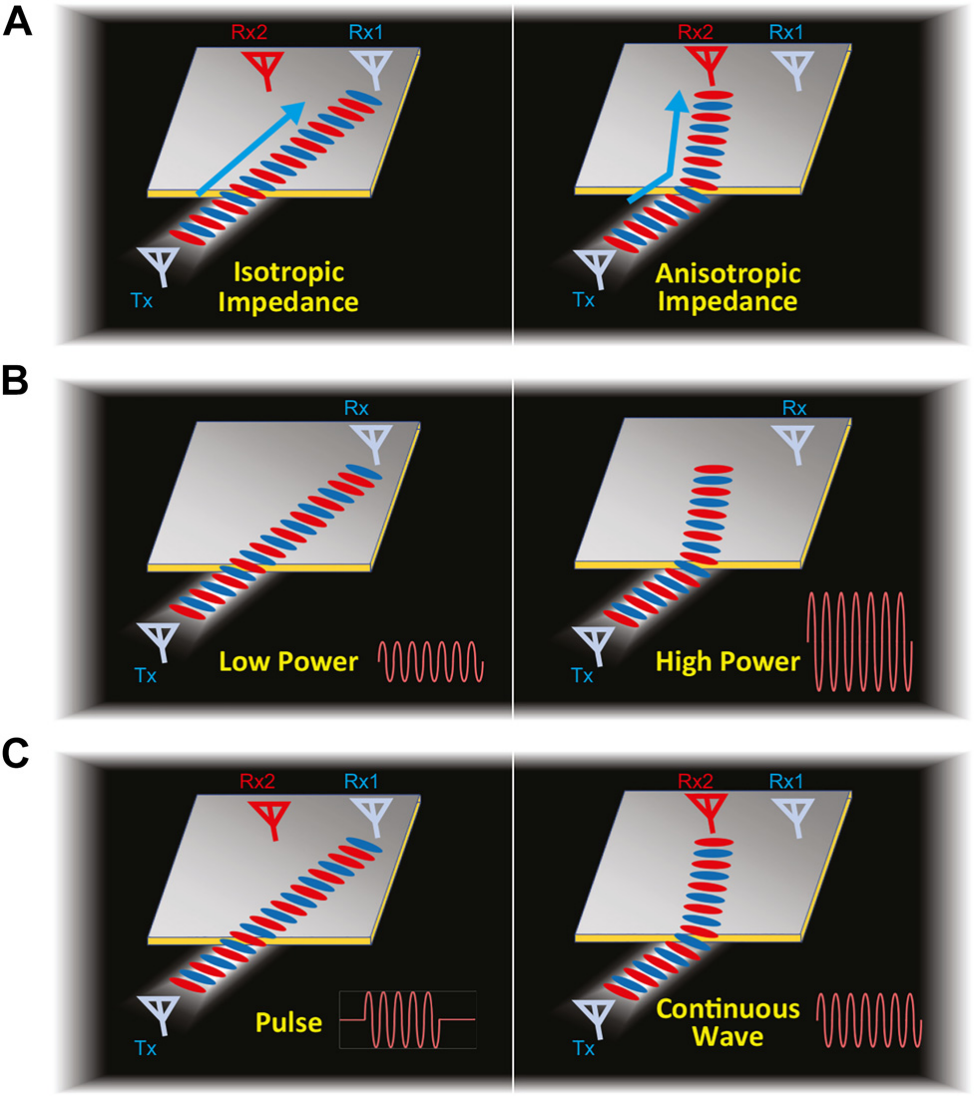
Isotropic and anisotropic surface response along with the applications of a switchable anisotropic impedance surface with power or waveform selectivity. (A) Isotropic and anisotropic impedance surfaces. The use of the anisotropic surface impedance leads to guiding an incoming surface wave along a designed direction. (B) Nonlinear switchable impedance surface. The surface has either an isotropic or anisotropic surface impedance (respectively, the left and the right) to maintain small-signal antenna communications while protecting a receiver antenna from high-power damaging surface waves at the same frequency. (C) More advanced switchable surface that, even with the same power level, still switches between an isotropic impedance (the left) and an anisotropic impedance (the right) in response to the type of an incoming waveform, namely, a pulse or continuous wave.

Early works on surface wave control have included engineering subwavelength structures with continuous changes in their permittivity or refractive index [11–13], which approximate methods used in metamaterials with transformation optics [14, 15]. Since surface waves only require 2D wave control, the structures are low-profile and much easier to fabricate than other 3D metamaterials [13]. In lower frequencies where ohmic losses are negligible, the relation between surface waves and their controllable medium can be expressed in terms of the surface impedance. Therefore, modeling surface waves with scalar and tensor impedance surfaces has been widely used [3, 16], [17], [18], [19], [20]. This method offers solutions to design controllable surface wave propagation by means of metallic layers patterned on top of dielectrics [21–23], thereby making various applications such as beam shifters [22], cloaking devices [24–27], and non-scattering waveguides [23]. In these structures, the anisotropic impedance surfaces exhibit a large difference in impedance between the two orthogonal directions, which may be exploited to launch and direct surface waves along an arbitrary path by rotating elements of the anisotropic unit cell (Figure 1A). Despite the high potential of engineered surface impedances, most studies are based on unit cells that have static properties once fabricated, limiting the control of surface waves only in a single fixed operation.

In recent works, nonlinear lumped elements have been added to the surface impedances to introduce tunability [28–30]. These nonlinear surface impedances have varying responses to the incoming surface waves depending on the power level (Figure 1B); thus, they are useful as a protection device in communication systems because they reject unwanted high-power signals from reaching receiver antennas [31]. In the literature, a nonlinear impedance surface was designed to realize self-focusing in the microwave domain [32]; this was equivalent to self-focusing nonlinear crystals for the optical regime. The device was realized using a mushroom structure with five vias in each unit cell and a complicated biasing of power sensing circuits. Other follow-up works have also been completed, such as realizing tunable far-field patterns from a reflective metasurface based on the incoming power level [33] and changing a transmission mode to a reflection mode through the use of a power-dependent meta-atom [34]. Despite the benefits of power-dependent devices, such a tunability may find many useful applications in the domain of electromagnetic compatibility (e.g., to suppress electromagnetic noise) or antenna design but less in the domain of wireless communications that use an almost constant level of signal strength for most practical modulation schemes [35, 36]. Therefore, an additional method to achieve the tunability of surface impedance is needed using the same power level. In more recent years, by introducing additional lumped elements such as resistors and inductors to control the transient response of incoming signals, a new tunability method based on waveform selectivity was reported [37, 38]. These nonlinear and transient surfaces are called waveform-selective surfaces which can distinguish between pulses and continuous waves. Such a unique tunability method has been exploited to realize switchable transmitters [39], signal-processing cavities [40], and intelligent absorbers [38] including the domain of wireless communications [41, 42]. However, waveform selectivity for surface wave control has so far been reported only for isotropic elements, and no elements of waveform-selective metasurfaces with anisotropic unit cells have been reported in the literature.

In this study, we propose switchable anisotropic impedance surfaces that can change the direction of surface wave propagation depending on the incident waveform type. We numerically validate the designed metasurfaces and analyze how surface wave propagation varies due to the presence of circuit embedded meta-atoms. To this end, firstly, the power-dependent impedance surfaces are designed using a pair of PIN diodes within each unit cell consisting of only one via, which is a much simpler configuration than previous nonlinear unit-cell elements. After the validation of the simple power-dependent surface, secondly, the waveform-selective impedance surfaces are designed using four diodes, a resistor, and an inductor in each unit cell; these components are configured as a nonlinear and transient circuit. Both circuit configurations are embedded within an array of mushroom unit cells providing the ability to switch between isotropic and anisotropic impedance surfaces. The main advantage of these switchable anisotropic impedance surfaces lies in the controllable steering of surface wave propagation by the incident power level or waveform type. Therefore, various interesting applications can be envisaged based on these new switchable anisotropic impedance surfaces. Firstly, interference from high-power incident beams in a receiver can be avoided by using a power-dependent surface impedance (see Figure 1B). Secondly, by using the anisotropic impedance surface with waveform selectivity, the selection of signals with a particular waveform can be realized; for example, this selectivity can separate pulses and continuous signals. Additionally, this waveform selectivity can be used to split receivers in wireless communication where two antennas receive different intended waveforms even at the same operational frequency (see Figure 1C). Such an operation is not possible to realize with current conventional filters or frequency-selective surfaces [43].

## 2 Anisotropic impedance surfaces

A conducting planar surface with a coating layer supports guided wave modes in which surface impedance may be used to characterize the entire surface. This surface impedance *Z* can be isotropic or anisotropic depending on the material and pattern being imposed. If we assume that the surface lies within the *x*–*y* plane, and consider a lossless and reciprocal case (i.e., *Z*_*xy*_ = *Z*_*yx*_), a tensor impedance boundary condition [21] may be used to represent the field relations within the surface:

(1) $$\left[\begin{array}{c} E_{x}\\ E_{y} \end{array}\right]=\left[\begin{array}{cc} Z_{xx} & Z_{xy}\\ Z_{xy} & Z_{yy} \end{array}\right]\left[\begin{array}{c} -H_{y}\\ H_{x} \end{array}\right]$$

where the impedance tensor is shown as a 2 × 2 matrix to associate electric field *E* and magnetic field *H*. This tensor impedance surface supports surface waves in transverse magnetic (TM) mode, transverse electric (TE) mode, and hybrid mode, which is a mixture of both the TM and TE modes. For a grounded dielectric the TM mode is fundamental and exists down to zero frequency. In an anisotropic case, the principal axes that are orthogonal to each other have a maximum and minimum phase velocity due to the minimum and maximum surface impedances. These surface impedances are equalized in the isotropic case where a uniform surface impedance applies. A more accurate and closed-form solution for this tensor impedance surface may be obtained by directly finding expressions for the direction of power flow [44].

**Figure 2:**
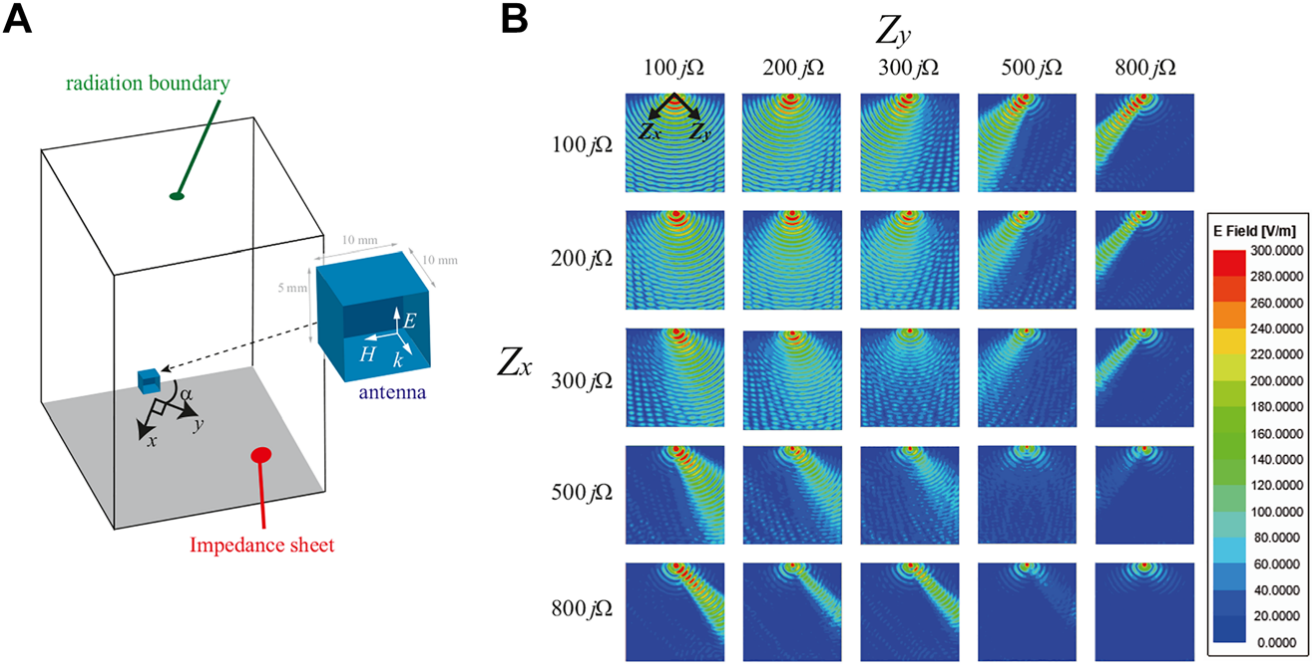
Simulation of surface wave propagation on an anisotropic impedance surface based on an impedance sheet model. (A) Simulation setup where *x* and *y* axes are fixed using *α* = 45°. (B) Electric field profiles for surface wave propagation on an anisotropic impedance surface with various impedances in the *x* and *y* directions. The surface impedances in the *x* and *y* directions vary as indicated by the variation in *Z*_*x*_ and *Z*_*y*_ determining how the surface wave propagates.

As passive static anisotropic impedance surfaces with the ability to shift beams into different directions have been well presented in the literature [22–27], here we focus on a method to introduce tunability into these artificial surfaces. In doing so, we consider a tunable surface that can be switched between an isotropic impedance and an anisotropic impedance. This switching mechanism is realized by controlling the surface impedances along the two principal axes; this method is equal to controlling their phase velocity and hence the direction of surface wave propagation. To analyze surface wave propagation on such an artificial surface, we use an impedance sheet in the commercial software ANSYS Electronics Desktop (2020 R2). An impedance boundary condition is assigned within the *x*–*y* plane in which *Z*_*xx*_ = *Z*_*x*_, *Z*_*yy*_ = *Z*_*y*_, and *Z*_*xy*_ = *Z*_*yx*_ = 0. As illustrated by the simulation setup in Figure 2A, these *x* and *y* axes have an *α* = 45° angle reference from one of the edges of the simulation box. Surface waves are excited by an antenna placed near the sheet to observe how TM surface waves propagate. Both the antenna and the impedance sheet are enclosed within radiation boundaries. The simulation results are shown in Figure 2B for various combinations of *Z*_*x*_ and *Z*_*y*_. It can be observed from the figure that when the impedances are equal in both orthogonal directions (i.e., *Z*_*x*_ = *Z*_*y*_), the surface is isotropic and the wave propagates without alteration, as shown by the diagonal plots from the top left to the bottom right of Figure 2B. When we separately change the impedance in the two orthogonal directions to introduce anisotropy, the surface wave is steered along the low impedance direction. In Figure 2B, we see that in the bottom-left plot where *Z*_*x*_ = 800 *j*Ω and *Z*_*y*_ = 100 *j*Ω, the wave is directed along the *Z*_*y*_ direction, while in the top-right plot, where *Z*_*x*_ = 100 *j*Ω and *Z*_*y*_ = 800 *j*Ω, the wave is directed along the *Z*_*x*_ direction. This confirms that by switching the isotropic and anisotropic conditions of the surface, wave propagation can be directed along certain paths. It is noted here that the assigned *Z*_*x*_ and *Z*_*y*_ are inductive reactance with positive values indicating that the surface wave propagation is in a TM mode.

**Figure 3:**
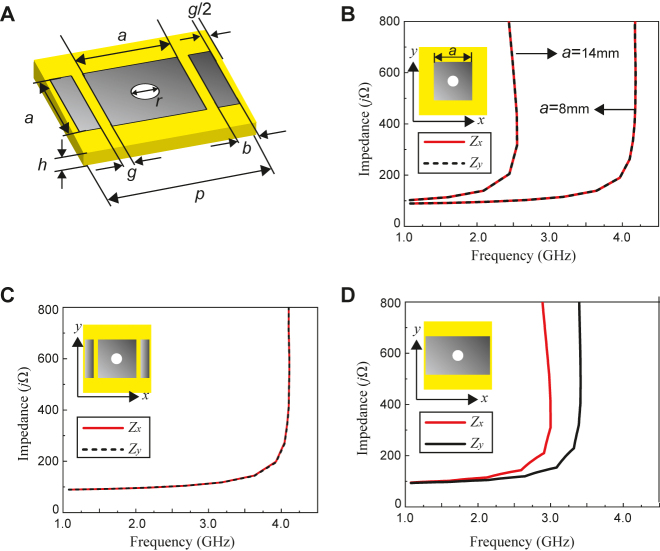
Details of unit cell structure and simulation results using eignemode solver. (A) Proposed unit cell structure consisting of a central square patch with a length of *a* connected with a via to the ground and with two rectangular side patches having a width of *b* placed nearby. The default dimensions are *g* = 1 mm, *p* = 15 mm, *a* = 8 mm, *b* = 2 mm, and *h* = 3 mm. (B)–(D) Simulation results of the unit cell structure using an eigenmode solver to extract the surface impedance. (B) is the square patch unit cell without the side patches (namely, isotropic condition). *a* is set not only to 8 mm but also to 14 mm. (C) is the square patch unconnected to the side rectangular patches (open-circuit, isotropic condition). (D) is the connected rectangular patch (short-circuit, anisotropic condition). The edge length of the top patch along *x* is *a* + 2*b* + 2*g* (i.e., 14 mm).

To realize such a switching mechanism based on more specific designs, a grounded dielectric substrate is used with a patterned metallic layer connected by vias, also known as mushroom structures [1]. The geometry of the proposed periodic unit cell is shown in Figure 3A. It is made of a double-sided printed circuit board (PCB) with a Rogers RO3003™ dielectric material, which has a permittivity of 3 and a loss tangent of 0.0013. The back side is a fully metallic ground plane while the unit cell of the front side consists of three patches including a square center patch that has a length of *a* = 8 mm. The gaps *g*s between the central patch and side patches are 1 mm. The side patch width is *b* = 2 mm. The periodicity *p* is 15 mm, and the dielectric thickness *h* is 3 mm. A via with a radius *r* = 0.5 mm is used to connect the bottom ground plane with the central patch. This unit cell is simulated using the eigenmode solver of ANSYS Electronics Desktop (2020 R2). A TM surface wave mode is considered in this design since the mode interacts favorably with the location of the lump components used later.

Using the above simulation setup, the surface impedances of the unit cell are extracted along two orthogonal directions, i.e., along *x* and *y*. This impedance extraction is based on dispersion curves obtained by the eigenmode solver, and the surface impedance is calculated by using the following formula [1, 23]:

(2) $$Z=\frac{j}{\omega \epsilon_{0}}\sqrt{k^{2}-\epsilon_{0}\mu_{0}\omega^{2}}$$

where *j* ^2^ = −1, *ϵ*_0_, *μ*_0_, *k*, and *ω* are, respectively, the permittivity and permeability of vacuum, wavenumber, and angular frequency. Three conditions are assumed in this simulation. The first condition is that a square central patch with a length of *a* is simulated without side patches (see the inset of Figure 3B). The second condition is that the square central patch has the side rectangular patches (the inset of Figure 3C). The third condition is that a connection is established between the central and side patches; thus, the entire conducting geometry forms one large rectangular patch (the inset of Figure 3D). It is known that for rectangular patches in a mushroom structure, i.e., with vias connected to the ground, strong anisotropic responses may be observed [21]. Figure 3B–D show the extracted impedances under the first, second, and third conditions, respectively. The results show that under the first condition, the impedances vary depending on the edge lengths of the top patch, although *Z*_*x*_ and *Z*_*y*_ have identical values due to the symmetric conducting design. Under the second condition, however, the impedances still remain almost identical for both *Z*_*x*_ and *Z*_*y*_, with no bandgap region before 4.0 GHz; this result indicates that the center square patch is a dominant factor to characterize the response of the entire surface, which appears as an isotropic surface impedance. In particular, the result for the unconnected patches is the same as that of the isotropic patch under the first condition with a length of *a* = 8 mm. It is also noted that below 4.0 GHz both *Z*_*x*_ and *Z*_*y*_ are lower than 200 *j*Ω in this configuration. Under the third condition using the connected rectangular patch that has a dimension of 14 mm by 8 mm, there is noticeable difference in the impedance profile in which *Z*_*x*_ rapidly increases near 2.8 GHz while *Z*_*y*_ remains well below 200 *j*Ω up until 3.2 GHz. This result indicates that below 2.8 GHz the longer the edge length is, the higher the surface impedance becomes. More importantly, the rectangular patch structure exhibits an anisotropic surface impedance. Due to this impedance difference in the *x* and *y* directions, the frequency region around 2.8 GHz permits the propagation of waves along the lower impedance direction instead of along the high impedance direction. In the following sections, we propose a unique method of switching between the conducting geometry of Figure 3C and that of Figure 3D, which can be utilized to demonstrate direction-dependent surface wave propagation-based applications.

It should be noted here that as shown in Figure 3B, the impedance *Z*_*x*_ has two values around 2.5 GHz, indicating two simultaneous TM modes (one for a forward mode and another for a backward mode) existing within a certain frequency region. Such a multi-valued impedance has also been discussed in previous studies of anisotropic impedance surfaces such as in Ref. [21], where it was highlighted that unwanted mode interference may arise within this specific bandwidth. However, this frequency region with multi-valued impedances is not used in the following part of our study. Our designed metasurfaces mainly exploit the bandgap region in the *x* direction (no propagation allowed) and the pass-band region where the impedance in *y* direction is very low (propagation allowed).

## 3 Power dependency and waveform selectivity

From the above unit-cell configuration, the key method that introduces tunability lies in changing the connection between the square central patch and the two side patches. Here, we introduce nonlinear circuit components that can determine the open or short state of the circuit depending on the power level or waveform type of the incident waves; this mechanism allows us to create impedance surfaces that demonstrate power dependency or waveform selectivity. In particular, the first case (i.e., power-dependent surface) is tested prior to the second case (i.e., waveform-selective surface) to show that the proposed switching mechanism works in a simpler case.

### 3.1 Metasurface with pairs of PIN diodes

**Figure 4:**
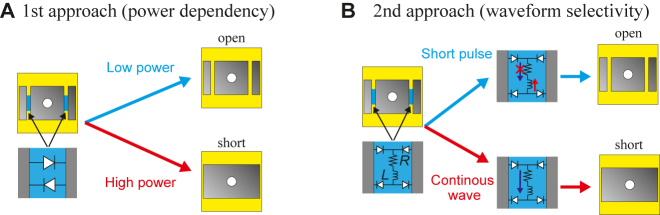
Mushroom unit cell with (A) a circuit containing a pair of PIN diodes for power dependency, and (B) an inductor-based circuit for waveform selectivity. Circuit values are given by *R* = 5.5Ω and *L* = 100 μH.

The first approach to implement tunability is to connect the square central patches with a circuit based on a pair of connected PIN diodes as shown in Figure 4A. By using this circuit, we essentially introduce nonlinearity to the unit cell based on the nonlinear properties of the diodes. In this configuration, when the incident wave induces electric charges on the patches, the diode circuit remains open if the incident energy level is low enough. However, when the energy level of the incident wave is increased, the induced potential difference can exceed the threshold voltage of the diode so that the circuit is shorted. For this reason, the diodes short the side patches with the central patch, and the entire conducting geometry forms a wide rectangular patch effectively. In this way, anisotropy is introduced in an otherwise isotropic patch, and the impedance response in two orthogonal directions becomes unique. The proposed design enables wireless control of surface wave propagation by varying the input energy level of the incident waves. Notably, no external biasing circuit is required to turn the diodes on and off, resulting in a much easier fabrication method than other metasurfaces with complicated control systems.

### 3.2 Metasurface with inductor-based circuits

In the second approach, tunability is achieved by using a transient circuit that can sense the type of the incident waveform, i.e., between pulses and continuous waves. Again, we short the side patches with the central patches to develop a single anisotropic structure. However, the circuit used here is based on the combination of a resistor and an inductor in series that is in a bridge configuration with four PIN diodes, as shown in Figure 4B. The main idea for the use of such a circuit is to exploit the frequency conversion of the incoming energy to zero frequency [37, 38, 41]. Here note that the diode bridge can fully rectify the incoming sine wave into the absolute form (|sin|) generating an infinite set of frequency components, although most of the energy is at zero frequency. For this reason, the incoming energy is then controlled by the inductor and resistor connected in series as seen in classic transient direct-current (DC) circuits. Due to the presence of the electromotive force of the inductor, the circuit acts as a high impedance for transient inputs such as pulse waves while it acts as a short circuit for steady-state signals such as continuous waves. In this way, a pulse wave opens the circuit and allows the patch to act as an isotropic structure characterized by scalar impedance, while a continuous wave shorts the circuit and connects the patches to act as an anisotropic structure having direction-dependent impedances along the two orthogonal axes.

## 4 Implementation and results

**Figure 5:**
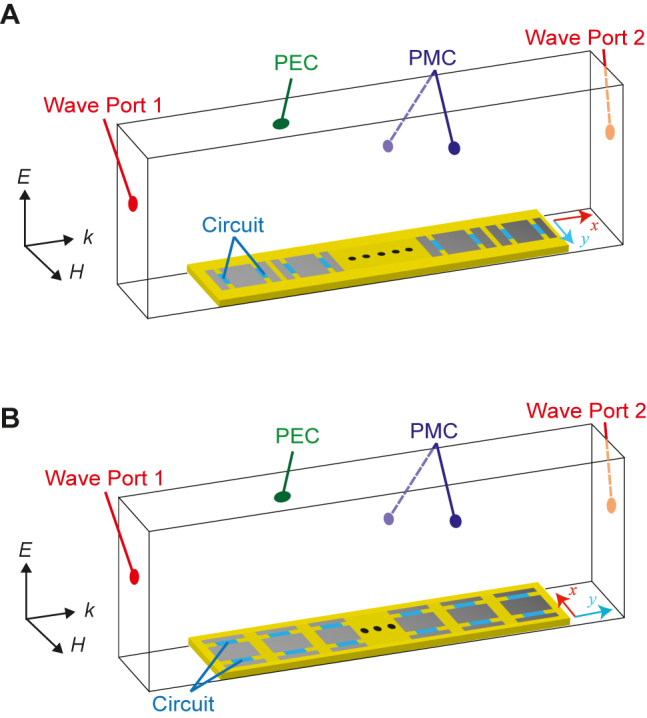
Surface wave propagation in a 1D model along two orthogonal directions: (A) *x*-direction and (B) *y*-direction. The lowest mode is excited by wave port 1, where the electric field is vertically polarized as drawn by the arrows of *E*. This mode effectively behaves as the lowest TM mode around the metasurface. The gaps between the metasurface and the wave ports are twice the periodicity (30 mm), which are larger than the quarter wavelength of 3.0 GHz (25 mm).

This proposed tunable unit cell that demonstrates power dependency or waveform selectivity is then tested for its surface wave propagation. The SMP1345 PIN diode model from Skyworks Solutions, Inc. is used in the circuit simulations. Ideally, a 2D model should be tested for the full visualization of the wave propagation of surface waves; however, we are limited to a 1D model to avoid the very large computational effort that would be needed due to the large number of diodes integrated in the surface. The 1D model is simulated in a waveguide configuration with perfect magnetic conductor (PMC) walls on the sides and perfect electric conductor (PEC) walls on the top and bottom and excited by a waveguide port in the longitudinal direction as shown in Figure 5. Similar to the eigenmode simulations (Figure 3) where we extracted the impedances along two orthogonal directions, here we test the surface wave propagation along the *x* and *y* directions. In the 1D configuration, this is performed by rotating each of the unit-cell patches to effectively simulate along the *x* and *y* directions, as shown in Figure 5A and B, respectively. In both cases, the number of the unit cells is set to ten. A co-simulation method is used in this 1D model, which integrates an electromagnetic simulator with a circuit simulator (see Figure S1 of the Supplementary Material in detail).

**Figure 6:**
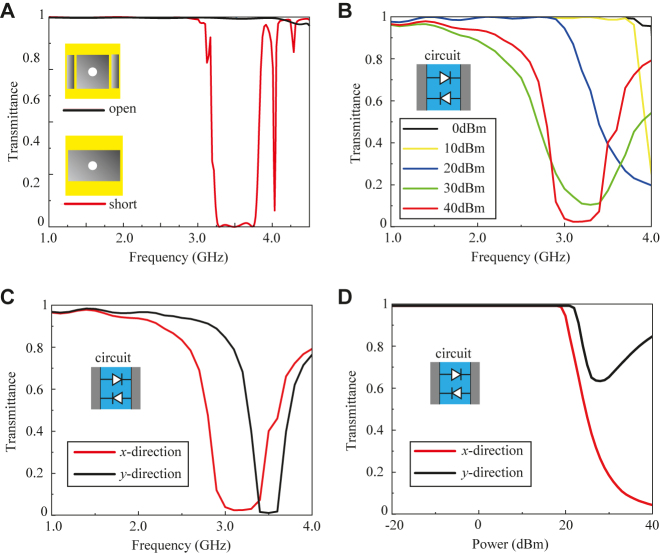
Surface wave propagation along the *x* direction (as in Figure 5A) with (A) open/short unit cells and (B) open unit cells connected by pairs of diodes at various power levels. (C) Validation of anisotropic surface impedance. Surface waves are set to propagate along the *x* direction or the *y* direction (as in Figure 5B) at 40 dBm. (D) Power dependency of the surface wave propagation along the *x* and *y* directions at 3 GHz. The input power is swept in 1 dBm steps.

The results from this 1D surface wave simulation for the unit cells with pairs of diodes are shown in Figure 6. Here, we firstly consider that the surface wave propagates along the *x* direction of the structure as in Figure 5A. As a reference, Figure 6A shows the transmittances of the structures used in Figure 3C and D. Both open and short patch structures are found to behave as low impedance surfaces and strongly transmit an incoming wave up to 3.0 GHz, although the short patch structure lowers the transmittance between 3.2 and 3.8 GHz. These are consistent with the results of Figure 3C and D. In Figure 6B, the open patch structure with paired diodes is then tested under various power levels from 0 to 40 dBm. When the incident wave has an input power of 0 dBm, the transmittance is at the maximum between 1 and 4 GHz. This is equivalent to the open patch structure in Figure 6A. On the other hand, when the incident power increases to 40 dBm in Figure 6B, the transmittance drops and reaches the minimum value between 2.8 and 3.2 GHz. This result matches that of the short patch model of Figure 6A. To validate the anisotropic impedance at a high incident power of 40 dBm, we monitor the transmittance of the surface wave along two orthogonal directions using the setups of Figure 5A and B. The simulation results for both cases are shown in Figure 6C. It can be seen that the transmittance is low when the surface wave propagates along the *x* direction within the frequency band of 2.8–3.2 GHz. In contrast, along the *y* direction, the transmittance is high within the considered bandwidth (2.8–3.2 GHz), although it decreases beyond 3.3 GHz as predicted by the second stop band of Figure 3D. These results imply that the structure has different impedances along two orthogonal directions within a specific bandwidth, thereby validating the anisotropic response of the structure.

Furthermore, to check the boundary between the isotropic and anisotropic surface impedances, we sweep the power level in finer steps at 3 GHz. The results are shown in Figure 6D, where it can be seen that for the propagation along the *x* direction (red curve), the transmittance is almost 100% up to an input power of 20 dBm, and then gradually decreases to the lowest transmittance at approximately 40 dBm. This result indicates that the impedance surface changes from a low impedance to a high impedance along the *x* direction due to the change in the circuit connecting the central patches, varying them from an open to a short state. In contrast, along the *y* direction, the transmittance is maintained above 60% with the variation in power level as shown by the black curve in Figure 6D. This relationship between the varying *Z*_*x*_ and the almost constant *Z*_*y*_ supports that the impedance surface changes from an isotropic impedance to an anisotropic impedance based on the power level.

**Figure 7:**
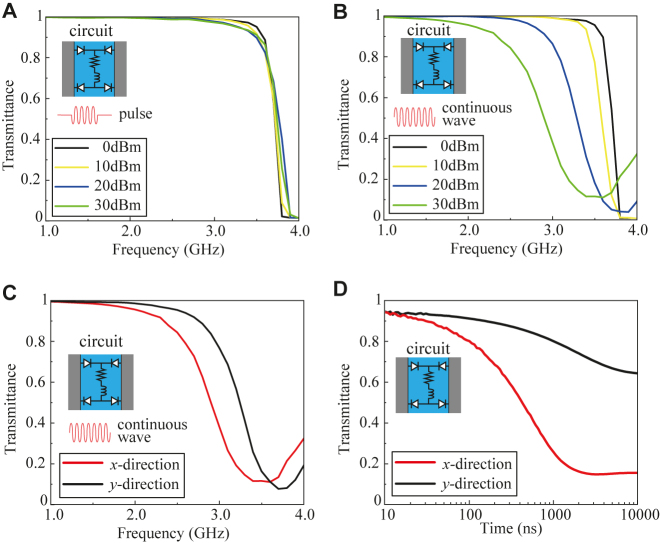
Surface wave propagation along the *x* direction (as in Figure 5A) at various power levels of (A) 50 ns pulse waves and (B) continuous waves. (C) Validation of anisotropic surface impedance. Surface waves are set to propagate along the *x* direction or the *y* direction (as in Figure 5B) for continuous waves at a power of 30 dBm. (D) Waveform selectivity of the surface wave propagation along the *x* and *y* directions at 3.0 GHz. The incident power is fixed at 30 dBm.

The other approach to achieve tunable impedance surfaces is based on a waveform-selective inductor-based circuit. As discussed in Subsection 3.2, we use sets of four diodes connected to the circuit containing an inductor *L* and a resistor *R* in series; additionally, we use *R* = 5.5 Ω and *L* = 100 μH for the components. The simulation with a 1D model along the *x* direction, as in Figure 5A, is performed, in which we use two waveform types for the surface waves, i.e., 50 ns pulse waves and continuous waves, at various power levels. The results for the 50-ns pulse waves are shown in Figure 7A where the transmittance is high at 3.0 GHz with all power levels from 0 to 30 dBm. On the other hand, for the continuous waves, different transmittance profiles are obtained as seen from Figure 7B. These results indicate that for the pulse waves, the impedance value is always at the minimum, while for the continuous waves, the impedance surface changes from low impedance to high impedance along the *x* direction due to the change in the circuits connecting the central patches (from an open to a short state). To validate that the structure behaves as an anisotropic impedance surface for this continuous wave with an input power of 30 dBm, we monitor the transmittance of the surface wave along two orthogonal directions using the setups shown in Figure 5A and B. The simulation results for both cases are shown in Figure 7C. It can be seen that the transmittance is low at 3.0 GHz when the surface wave propagates along the *x* direction, as plotted by the red curve. In contrast, along the *y* direction, as shown by the black curve, the transmittance is high at 3.0 GHz, although the difference between both curves decreases beyond 3.3 GHz. Nevertheless, these results imply that the structure has different impedances along two orthogonal directions at 3.0 GHz, validating the anisotropic response of the structure.

To check the boundary between the isotropic and anisotropic surface impedances with respect to the pulse width of the incoming wave, we conduct a time-domain analysis in the 1D simulation setup along both the *x* and *y* directions at 3.0 GHz. The results are shown in Figure 7D where for the propagation along the *x* direction (red curve), the transmittance is above 50% up to a pulse width of 700 ns, and then gradually decreases to the lowest transmittance when above a pulse width of 1000 ns. This result indicates that the impedance surface changes from a low impedance to a high impedance along the *x* direction due to the change in the circuits when connecting the central patches (from an open to a short state). In contrast, along the *y* direction, the change in the transmittance is relatively limited with an increase in the pulse width, as drawn by the black curve in Figure 7D. From the two curves in Figure 7D, the proposed structure is found to have an isotropic impedance during an initial time period or for short pulses, which is switched to an anisotropic impedance at a steady state or for continuous waves.

## 5 Discussion

Although our structures may resemble the ones used in the literature [37], this study had three distinctive differences that enabled us to realize the switchable anisotropic surface impedance. Firstly, any of the past studies deployed transient circuits in the gaps between conductor edges where electric field was strongly concentrated to *absorb* the energy of the incoming wave in response to the waveform type. In this study, however, the circuits were used to *re-construct* periodic conducting geometries. Secondly, the use of conducting vias was necessary for designing a significant change in the frequency dependence of the surface impedance. In other words, the proposed structures without vias varied the surface impedance but with a gentle increment, which did not successfully produce a clear contrast between surface impedances for two orthogonal directions. Thirdly, the switching mechanism in the metasurface presented here enabled steering of surface wave by the incident waveforms or power levels, while the previously reported metasurfaces only changed the magnitude of absorptance. There have been many works on nonlinear waveguide couplers depending on the transmitted power in optics [45, 46]. However, none of these studies has exploited the transient response that depends on incident waveform types.

As illustrated in Figure 5, 1D simulations of switchable anisotropic metasurfaces were conducted to validate their operations. These simulations were used since the full 2D simulations required more computational resources than realistically available due to a huge number of nonlinear components. Nevertheless, we do not expect that 2D simulations show a significant difference from the 1D simulation results since the use of PMC boundary condition in 1D simulations is effectively equivalent to evaluating an infinite array of unit cells. However, Figure S2 in Supplementary Material provides 2D simulation results where our metasurfaces were simplified to be linear structures. The simulation results support that the surface wave steering using the proposed metasurfaces is realizable for applications such as splitting receivers in wireless communication as illustrated in Figure 1C.

In the 2D simulations in Figures 2 and S2, open-ended waveguides composed of PECs and PMCs were used to excite surface waves, which were fed using wave ports. In the experiment to generate TM-mode surface waves within the microwave range, several surface wave launchers are commonly used such as Yagi-Uda like antennas [47], flared microstrips [48], or trapezoidal launchers [23]. A simple monopole antenna can also serve as a TM-mode surface wave launcher when placed on top of the metasurface [1]. Also, free-space waves can be converted to surface waves by phase-gradient metasurfaces [49].

It is noted here that design parameters used in this study have been carefully chosen to allow direct translation into practical experimental design. The applicability of the proposed metasurfaces has also been evaluated by comparing to our earlier experimental results, where we verified a waveform-selective metasurface working as an absorber for short communication signals [42]. In the experiment of Ref. [42], a waveform selectivity (i.e., a clear difference between absorptance for a short pulse and that for a continuous wave) was maximized when the input power was 10 dBm or so, while this study required 30 dBm (see Figure 7C) due to the use of a different diode model (SMP1345 PIN diode). 30 dBm is much larger than the power level of ordinary communication signals but is not unrealistic to protect a sensitive electronic device from a high-power source [50–52], as explained in Figure 1B. To realistically exploit waveform-selective anisotropic surface impedance, the operating power can be lowered by using ordinary semiconductor products [53]. Moreover, the use of advanced semiconductor technologies also contributes to markedly reducing the operating power level. For instance, the use of down-scaled semiconductor process leads to shrinking a die size and to lowering the operating power of a diode [54, 55]. Waveform-selective mechanisms themselves have been experimentally validated in our previous studies [41, 56, 57]. However, as we focused on the concept validation in this study, we did not conduct any optimization to lower the operating power of the diodes used but instead adopted an SPICE model of the commercial product (i.e., SMP1345 PIN diode). This topic along with the experimental validation remains as a future follow-up work. Also, the design of the proposed metasurfaces can be further improved by introducing inhomogeneous cells or an aperiodicity [58–60].

## 6 Conclusions

In this paper, we have demonstrated switching mechanisms between an isotropic impedance surface and an anisotropic impedance surface. These mechanisms are achieved by loading unit cells of a mushroom structure with two different circuits that can change between an open state and a short state depending on the incident power level or waveform type. The circuit consisting of a pair of diodes is switchable by the power level of the incident waves while the circuit consisting of a resistor and an inductor in series as well as four diodes in a bridge configuration is switchable based on the waveform type of the incident waves. The switching from an isotropic to anisotropic impedance surface results in different levels of transmittance along two orthogonal directions, eventually causing the surface wave to only propagate along the direction of the lower impedance. The results obtained through numerical simulations based on a 1D configuration of the unit-cell arrays validate the proposed design approach. The switchable anisotropic surface impedance proposed here is a promising platform for various functionalities such as coupling and isolation of antenna elements, cloaking, sensing, wireless charging, and communications.

## Supplementary Material

Supplementary Material Details

Supplementary Material Details
