# Supplementary material for: Anisotropic impedance surfaces activated by incident waveform
Source: Nanophotonics. 2022 Feb 7;11(9):1989–2000. doi: 10.1515/nanoph-2021-0659 (PMC11501885; doi:10.1515/nanoph-2021-0659)
Supplement: Supplementary file 1 — Supplementary Material Details [file j_nanoph-2021-0659_suppl_001.docx]

Supplementary Material

**Anisotropic Impedance Surfaces Activated by Incident Waveform**

Haruki Homma, Muhammad Rizwan Akram, Ashif Aminulloh Fathnan, Jiyeon Lee, Christos Christopoulos, and Hiroki Wakatsuchi*

*e-mail: wakatsuchi.hiroki@nitech.ac.jp

1. Simulation Method
   1. Co-simulation for 1D Modeling of Surface Impedances


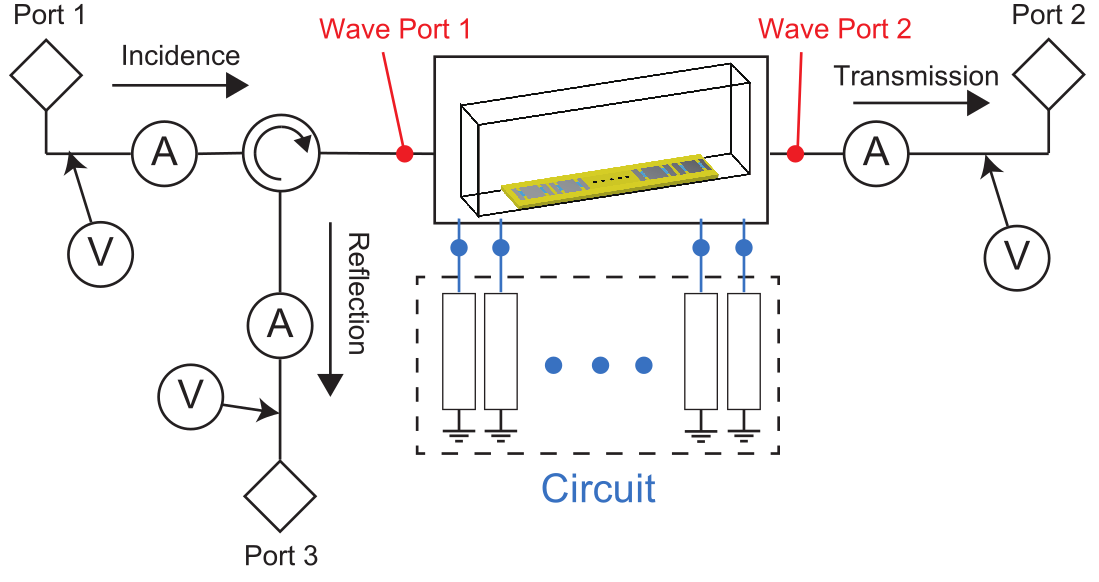


**Fig S1.** Co-simulation method where electromagnetic simulation was integrated with circuit simulation inside ANSYS Electronics Desktop (2020 R2).

To analyze the proposed metasurfaces, we used a co-simulation method that integrated an electromagnetic simulator with a circuit simulator (ANSYS Electronics Desktop (2020 R2)). In this method, we first simulated a 1D metasurface consisting of ten unit cells arranged across either a *x* or *y* direction and conducted analysis using HFSS electromagnetic simulator. PMC and PEC boundary conditions were used as explained in the main text. After obtaining the electromagnetic analysis results, we created a dynamic link to make the electromagnetic simulation results accessible in the circuit simulator. Here we connected circuit components including PIN diodes to lumped ports as designed in Fig. 4 of the main text. Other components were also used to generate short pulses or continuous waves and to monitor incident, reflected, and transmitted voltages (V) and currents (A) as shown in Fig. S1. To conduct circuit simulation, either a transient solver (for pulses) or a harmonic balance solver (for continuous waves) was used. Transmittance, reflectance, and absorptance were calculated in this circuit simulation, and overall simulation time was markedly reduced in this method, compared to ordinary electromagnetic simulation methods.

- 1. Realistic simulation using 2D Model

**
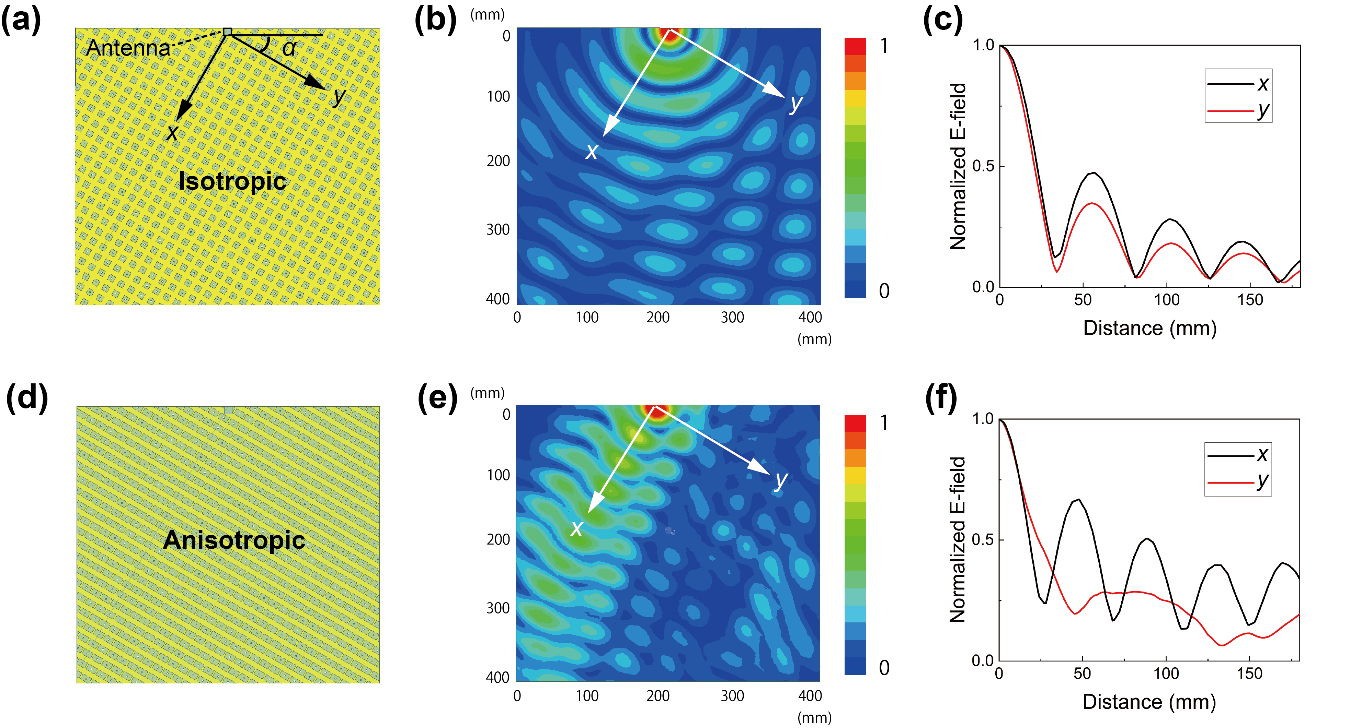
**

**Fig S2.** Isotropic and anisotropic metasurfaces and their normalized electric field profiles. Frequency was set to 3.5 GHz.

The metasurfaces made of passive patches were simulated using full wave analysis, and the results are shown in Fig. S2 for both isotropic and anisotropic cases. Note that here we only used linear conducting patches that were simplified to be equivalent to the ones including nonlinear circuit components in the main text. This was simply because the full wave analysis including nonlinear circuits required much more computational resources than realistically available. Therefore, the absence of nonlinear characteristics potentially makes a difference in the simulation results of Fig. S2. Nonetheless, since PIN diodes can be briefly approximated by a short circuit or an open circuit, as seen in existing studies [1-4], still a similar guiding performance can be expected in the case of metasurfaces loaded with nonlinear components. Firstly, the isotropic metasurface used here was modeled as square patches (8 mm by 8 mm with a 15-mm periodicity) with sides aligned along *x* and *y* directions while for the anisotropic case, the square conductors were replaced with rectangular patches (13 mm by 8 mm) such that longer edges were aligned along *y* axis. Both cases used vias (3 mm tall) connecting the patches to the ground metallic layer. To simplify the coordinate interpretation, we set up *x* and *y* axes to make an angle *α*=30^o^ from the horizontal line of Fig. S2a. The waveguide source (10 mm in width, 5 mm in height, and 10 mm in depth) was used slightly above the metasurface to efficiently generate the lowest TM surface wave mode. The results are shown in Fig. S2 for full 2D field profiles (Figs. S2b and e) and 1D field profiles (Figs. S2c and f). For the anisotropic case, since the low impedance direction was along *x* axis, the surface wave mostly traveled along the *x* direction instead of the *y* direction where impedance was higher (Fig. S2d). For the isotropic case, however, the wave quickly spread out away from the incident source, and there was not much contrast for the two orthogonal directions (Fig. S2b). Moreover, the decay rate was higher for the isotropic case, compared to the anisotropic case as plotted by the 1D field profiles of Figs. S2c and f. The clear contrast of the surface wave propagation for the two orthogonal directions is important to utilize the anisotropy for various surface wave steering applications.

**References**

1. S. Sun, W. Jiang, S. Gong, and T. Hong, “Reconfigurable linear-to-linear polarization conversion metasurface based on PIN diodes,” *IEEE Antennas Wirel. Propag. Lett.*, vol. 17, no. 9, pp. 1722-1726, 2018.
2. J. Tian, X. Cao, J. Gao, et al., “A reconfigurable ultra-wideband polarization converter based on metasurface incorporated with PIN diodes,” *J. Appl. Phys.*, vol. 125, no. 13, p. 135105, 2019.
3. J. Wu, Z. Zhang, X. Ren, et al., “A broadband electronically mode-reconfigurable orbital angular momentum metasurface antenna.” *IEEE Antennas Wirel. Propag. Lett.*, vol. 18, no. 7, pp. 1482-1486, 2019.
4. M. Liu, A. B. Kozyrev, and I. V. Shadrivov, “Time-varying metasurfaces for broadband spectral camouflage,” *Phys. Rev. Appl.*, vol. 12, no. 5, p. 054052, 2019.
